# To what extent do food purchases reflect shoppers’ diet quality and nutrient intake?

**DOI:** 10.1186/s12966-017-0502-2

**Published:** 2017-04-11

**Authors:** Bradley M. Appelhans, Simone A. French, Christy C. Tangney, Lisa M. Powell, Yamin Wang

**Affiliations:** 1 Department of Preventive Medicine, Rush University Medical Center, 1700 W. Van Buren St., Suite 470, Chicago, IL 60612 USA; 2 Department of Behavioral Sciences, Rush University Medical Center, 1645 W. Jackson Blvd. Suite 400, Chicago, IL 60612 USA; 3 Division of Epidemiology and Community Health, School of Public Health, University of Minnesota, 1300 S 2nd Street, Suite 300, Minneapolis, MN 55454 USA; 4 Department of Clinical Nutrition, Rush University Medical Center, 1700 W. Van Buren St., Suite 425, Chicago, IL 60612 USA; 5 Health Policy and Administration, School of Public Health, University of Illinois at Chicago, MC 923, 1603 W Taylor St., Chicago, IL 60612 USA; 6 Department of Internal Medicine, Rush University Medical Center, 1645 W. Jackson, Suite 675, Chicago, IL 60612 USA

**Keywords:** Food purchasing, Dietary assessment, Diet quality, Energy density, Healthy eating index

## Abstract

**Background:**

Food purchasing is considered a key mediator between the food environment and eating behavior, and food purchasing patterns are increasingly measured in epidemiologic and intervention studies. However, the extent to which food purchases actually reflect individuals’ dietary intake has not been rigorously tested. This study examined cross-sectional agreement between estimates of diet quality and nutrient densities derived from objectively documented household food purchases and those derived from interviewer-administered 24-h diet recalls. A secondary aim was to identify moderator variables associated with attenuated agreement between purchases and dietary intake.

**Methods:**

Primary household food shoppers (*N* = 196) collected and annotated receipts for all household food and beverage purchases (16,356 total) over 14 days. Research staff visited participants’ homes four times to photograph the packaging and nutrition labels of each purchased item. Three or four multiple-pass 24-h diet recalls were performed within the same 14-d period. Nutrient densities and Healthy Eating Index-2010 (HEI-2010) scores were calculated from both food purchase and diet recall data.

**Results:**

HEI-2010 scores derived from food purchases (median = 60.9, interquartile range 49.1–71.7) showed moderate agreement (ρc = .57, *p* < .0001) and minimal bias (-2.0) with HEI-2010 scores from 24-h recalls (median = 60.1, interquartile range 50.8–73.9). The degree of observed bias was unrelated to the number of food/beverage purchases reported or participant characteristics such as social desirability, household income, household size, and body mass. Concordance for individual nutrient densities from food purchases and 24-h diet recalls varied widely from ρc = .10 to .61, with the strongest associations observed for fiber (ρc = .61), whole fruit (ρc = .48), and vegetables (ρc = .39).

**Conclusions:**

Objectively documented household food purchases yield an unbiased and reasonably accurate estimate of overall diet quality as measured through 24-h diet recalls, but are generally less useful for characterizing dietary intake of specific nutrients. Thus, some degree of caution is warranted when interpreting food purchase data as a reflection of diet in epidemiological and clinical research. Future work should examine agreement between food purchases and nutritional biomarkers.

**Trial registration:**

ClinicalTrials.gov, NCT02073643. Retrospectively registered.

## Background

Diet quality is associated with heightened risk for all-cause mortality, cardiovascular disease, type II diabetes, and cancer [1, 2], and the role of food purchasing patterns as a driver of diet and chronic disease risk is a growing area of research. Studies conducted to date support several broad conclusions about food purchasing patterns. First, the healthfulness of household food purchases follows a socioeconomic gradient. Individuals with lower household incomes or less education tend to purchase calories in less expensive forms (i.e., lower dollars per calorie) that are less nutrient-rich [3–6]. This pattern of socioeconomic differences in food purchases is also apparent “downstream” in dietary intake [7] and is thought to contribute to disparities in chronic disease risk [8]. A second consistent finding in the literature is that the nutritional value of purchased foods varies greatly across different food sources. Fast food and carryout restaurants, and small food stores such as corner stores, gas stations, and dollar stores, are major sources of packaged snacks and sugar-sweetened beverages [9] and generally offer foods associated with lower diet quality [10–12]. In addition to observational research, studies have demonstrated that food purchasing is modifiable through intervention. Specifically, improvements in purchasing of healthful foods have been observed with interventions consisting of promotion/advertising of healthy items, point-of-purchase nutrition counseling, pricing interventions, and monetary incentives [13–16]. Several corner store interventions that focused solely on improving the availability of healthful foods have produced inconsistent effects on purchasing patterns [17–19].

Several methods have been used to measure food purchasing patterns, with each approach providing information with different levels of granularity. A number of studies have analyzed grocery receipts in order to tabulate food expenditures (in currency units) within different categories (e.g., sugar-sweetened beverages, fruits and vegetables, packaged snacks) [20–25]. Using this approach, French et al. [22] determined that 2 weeks of receipt collection was sufficient to adequately estimate household food purchasing patterns. It is also possible to obtain detailed estimates of the nutrient content of food purchases by pairing purchase data from individual research subjects with nutrition information for purchased products. Detailed information on the type, brand, and amount of each purchased product can be documented through digital photography [3] or the use of a handheld barcode scanner [26–28], and nutrition information for each product can then be extracted from commercially available nutrition analysis software or a retailer’s proprietary database. Large consumer-driven databases (e.g., Nielsen’s National Consumer Panel [26], USDA National Household Food Acquisition and Purchase Survey [29]) include both expenditure and nutrition data for purchases scanned by panelists after each shopping trip. These databases include purchases from multiple sources, but their reliance on potentially unreliable user-driven documentation methods is a key limitation [26].

Though it is logical to presume that food purchases are an accurate proxy for dietary intake (i.e., that people eat what they buy), this assumption has not been rigorously tested. The relative contributions of away-from-home foods, food waste, and consumption by other household members may each affect the degree to which episodes of food purchasing reflect actual dietary intake. Only two research groups have compared the nutrient content of food purchases and dietary intake. Ransley et al. [30, 31] derived the energy and fat content of food purchases using receipts collected by UK supermarket shoppers, and Eyles et al. [32] trained 49 New Zealand adults to record their supermarket purchases with a handheld scanner. Strong correlations between nutrient estimates from food purchases and reported dietary intake were observed in both of the aforementioned studies. However, only purchases from a single supermarket were examined.

The present study sought to determine whether household food purchases could be used to accurately estimate diet quality and nutrient intake in adults who purchase the majority of foods for their household. Packaged and non-packaged food purchases from all food sources were documented through a protocol that combined receipt collection and annotation with digital photography. It was hypothesized that nutrient densities and Healthy Eating Index-2010 diet quality scores derived from 2 weeks of household food purchases would demonstrate agreement with estimates calculated from three contemporaneous 24-h diet recalls. The degree to which agreement varied with key participant characteristics (e.g., social desirability, body mass, household size and income) was examined.

## Methods

### Participants

**Fig. 1:**
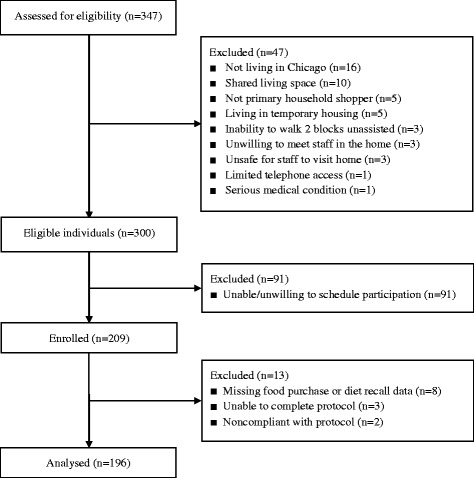
Flow of participants through the study

The sample was composed of Chicago households enrolled in the Study of Household Purchasing Patterns, Eating, and Recreation (SHoPPER), a cross-sectional study of behavioral and socioeconomic correlates of food purchasing patterns (ClinicalTrials.gov identifier: NCT02073643). A convenience sample was recruited from the community between 2014 and 2016 through posted flyers, newspaper advertisements, mailings, craigslist.org, word-of-mouth, and other methods. Interested individuals completed a telephone screening to assess eligibility. Adults who reported making ≥75% of their household’s food purchases were eligible to participate. Exclusion criteria included: 1) non-fluent in English, 2) not living in Chicago, 3) major food allergies or sensitivities, 4) religious/spiritual or medical dietary restrictions that could impact food choice (e.g., upcoming religious fasts), 5) living in temporary or group housing or living with a roommate with whom food is shared, 6) lack of telephone/cell phone access, 7) inability to walk 2 blocks unassisted, 8) serious medical conditions that interfere with daily life (e.g., kidney disease on dialysis), 9) history of psychotic disorder, eating disorder, or syndromal cause of obesity, 10) unwilling to meet with researchers in their home, and 11) conditions that would make it unsafe for researchers to visit their home (e.g., criminal activity near the home, extreme unsanitary conditions). Of 347 households screened, 300 (86.5%) met eligibility criteria and 209 (69.7%) ultimately scheduled their participation and enrolled (Fig. 1). Five participants were withdrawn from the study because of scheduling conflicts that arose during the 14-d assessment period (*n* = 3) or due to noncompliance with the protocol (*n* = 2). The analytic sample includes 196 subjects with complete food purchase, diet recall, and sociodemographic data. Participants were compensated $100 for completing all four assessments. Written informed consent was obtained from all participants. Study procedures were approved by the Rush University Medical Center Institutional Review Board.

### Procedures

#### Data collection procedures

All data were collected by trained research staff who visited participants’ homes four times during a 14-d assessment period. The final visit always occurred on the 14th day of the assessment period. Twenty-four hour diet recalls were performed by a masters-level dietitian at three of the four visits, with at least one recall capturing weekend dietary intake. Participants documented their household food purchases throughout the 14-d assessment period using the protocol described below. Research assistants telephoned participants between visits to answer questions and promote compliance to the protocol. To obtain a representative characterization of purchasing patterns, data collection did not occur within 1 week of major U.S. holidays or other religious holidays celebrated by participants.

#### Food purchase documentation

The primary household food shopper was trained to collect and annotate food receipts from all household members on a daily basis. Researchers provided a binder containing step-by-step instructions, and food purchase documentation forms that were to be completed for all food purchases (even for purchases without a receipt). Documentation forms captured information on the food purchasing occasion (i.e., date, time, source type and location, payment methods, and shopper name), and foods purchased within that occasion (quantity, size, price, and brief description). Participants were instructed to apply matching color-coded and/or numbered stickers to the documentation form, the receipt itself, and to all of the foods and beverages listed on that form. A different set of stickers was to be used for each purchase occasion so that research staff could easily match foods with the appropriate receipt and documentation forms. Food packaging contains important information needed to accurately identify each product, including the brand, variety, and amount contained in each package.Therefore, after consuming foods purchased during the assessment period, participants were asked to save all non-perishable food packaging in a large paper bag labeled with the study logo.

At each home visit, research staff collected food receipts and documentation forms, and clarified any ambiguous entries with participants. Staff then located all purchased foods or saved packaging in the home based on the affixed stickers, and digitally photographed the packaging and nutrition label of each item. For purchases without packaging or nutrition labels (e.g., fresh produce, deli items, bulk nuts/candy), researchers took field notes identifying the specific type of food and amount purchased. For foods purchased in ready-to-eat form and consumed immediately (e.g., carry-out or restaurant meals), research staff queried participants for information on portion size, preparation method, and key ingredients. Ready-to-eat foods that could not be accurately characterized (e.g., a buffet meal purchased and consumed by a household member other than the primary shopper) were deemed “non-codable” and were not subjected to nutrient analysis (<1% of all purchases).

#### Data management and nutrient analysis

Purchase data were stored in three separate data tables. First, descriptive information on each purchasing occasion (from the food purchase documentation forms) was entered into a relational database via MS Access. Second, the nutrient content of each purchased food was derived using the Nutrition Data System for Research (NDSR: versions 2013–2015, Nutrition Coordinating Center, University of Minnesota, MN), which contains nutrient information for over 18,000 foods. Research staff relied on digital photographs and field notes taken during home assessment visits to accurately identify each purchased item within the NDSR database. Nutrient data were obtained for the entire amount of each food purchased. Purchases were organized within NDSR by purchasing occasion, and exported into a table. Entries in this SQL table populated a Microsoft Access data entry form that research staff used to append price data to nutrient data for each purchased item (price data not reported). This data management process yielded a large dataset in which the nutrient and price data for each item was nested within purchasing occasions and individuals.

### Measures

#### Demographic and social variables

The primary shopper reported their age (derived from date of birth), gender, ethnicity/race, educational attainment, employment status [unemployed, work disability, part-time (<35 h/week), full-time (≥35 h/week), retired] household size and composition, current tobacco use, and household income (reported to the nearest $100/y). The income to poverty ratio was calculated by dividing annual household income by the current Federal Poverty Threshold [33], which accounts for the number of adult and child family members in each household.

#### Anthropometrics

Height and weight of the primary shopper were measured in light clothing and stocking feet using a portable electronic stadiometer (model 213) and flat scale (model 876) from SECA (Hamburg, Germany). Body mass index (BMI) was calculated as weight (kg) divided by height^2^ (m).

#### Dietary intake

Dietary intake was assessed for primary shoppers through three in-person, 24-h diet recalls conducted at home visits. Recalls captured intake on two non-consecutive weekdays (M = 2.1, SD = 0.4 weekday recalls per subject) and one weekend day (M = 1.0, SD = 0.2 weekend recalls per subject), which were ultimately averaged. Diet recalls were guided by the NDSR diet recall software, which is modelled after the USDA interviewer-administered Automated Multiple-Pass Method. A booklet containing two-dimensional illustrations of various foods, known as the Food Portion Visual™ [34], was referenced to facilitate portion size estimation. Recalls were conducted by research dietitians with formal training in diet recall methodology, and all recalls were reviewed by a nutrition epidemiologist (CCT).

#### Nutrient densities and diet quality of purchases and recalled intake

The densities of fruits, vegetables, and seven key nutrients per 1000 kcal of food were calculated for both food purchases and recalled dietary intake using identical methods. Energy density was calculated in units of kcal/g. Additionally, the Healthy Eating Index-2010 scoring system [35] was applied to nutrient data derived through NDSR in order to quantify overall diet quality of purchased (HEI-2010_purchased_) and consumed (HEI-2010_consumed_) foods. The HEI-2010 scores the nutrient densities (per 1000 kcal) for 11 of 12 key dietary components on a continuous scale based on conformity to the Department of Health and Human Services’ 2010 Dietary Guidelines for Americans [36], The twelfth score is based on the ratio of monounsaturated and polyunsaturated fatty acids to saturated fatty acids. The 12 component scores are summed to obtain a total score with a maximum of 100 points, with higher scores reflecting better overall diet quality.

Medications, nutritional supplements, infant formulas, baby foods, and chewing gums were excluded from calculations of diet quality and nutrient densities. We also explored the impact of three alternative scoring methods on HEI-2010_purchased_ estimates and their concordance with HEI-2010_consumed_: 1) excluding beverages and beverage mixes, 2) excluding cooking and baking ingredients, and 3) truncating each food to a maximum of 5000 kcal to reduce the impact of very large food purchases.

#### Time spent documenting food purchases

At each assessment visit, participants indicated how much time they had spent collecting and annotating food purchase receipts since the prior visit three to four days earlier.

#### Social desirability

Social desirability refers to a tendency to present one’s self in a manner consistent with perceived social norms. Participants in the present study completed “Short Form C” of the Marlowe-Crown Social Desirability Scale [37], which includes 13 true-false items from the original 33-item scale. Higher scores indicate greater social desirability. Greater social desirability has been associated with inaccurate self-reporting of dietary intake in prior studies [38, 39], and was considered a potential influence on concordance between food purchases and 24-h diet recalls in this study.

### Statistical analyses

Analyses were performed using Stata 13.1 (College Station, TX). Descriptive statistics were calculated to characterize the study sample and food purchasing variables. Variable distributions were examined for normality and extreme values using skew and kurtosis indexes and normal quantile plots.

For descriptive purposes, median values and interquartile ranges were reported for food purchase data both overall and by food source. The reported food purchase data include the number of receipts collected by the participant, the number of line item food purchases (i.e., distinct purchases listed on each receipt), total food mass and energy purchases, HEI-2010 scores, and nutrient densities. These values were calculated for all food purchases combined, as well as food purchases from different food sources.

To examine agreement between food purchases and diet recalls, HEI-2010 scores and nutrient densities from both methods were compared using Lin’s concordance correlations (ρc) [40], which assesses agreement between measures as a function of the Pearson correlation and the deviation of their best-fit line from perfect concordance. Additionally, the Bland-Altman limits-of-agreement method [41] was used to determine the extent to which HEI-2010_purchased_ provides an unbiased estimate of HEI-2010_consumed_ across the range of observed scores. The Bland-Altman method quantifies bias as the average difference in the estimates (bias) provided by two measures with the same measurement scale, and provides a 95% confidence interval for this difference.

Additional analyses sought to identify moderators of agreement between estimates of diet quality derived from food purchases and 24-h diet recalls. For each participant, the difference between HEI-2010_purchased_ and HEI-2010_consumed_ was calculated, as well as the absolute value of this difference. Spearman correlations (ρ) tested associations between these difference scores and potential sources of systematic bias and error, including income to poverty ratio, body mass index, household size, number of reported food purchases, and social desirability. Lowess curves were plotted to determine if agreement between HEI-2010_purchased_ and HEI-2010_consumed_ demonstrated a non-linear association with number of reported food purchases.

## Results

Diet recall and food purchase data were available from 196 primary household food shoppers, the majority of whom were female (*n* = 163, 83.2%). Sociodemographic characteristics and social desirability scores are shown in Table 1, as are descriptive data on diet quality (HEI-2010_consumed_ scores), energy intakes and food/nutrient densities based on 24-h diet recall data.

**Table 1:** Characteristics of 196 primary household food shoppers

	*Mean (SD)*
Age (y)	44.0 (13.2)
Household members (*n*)	2.5 (1.6)
Social desirability score (0–13 possible) ^a^	8.3 (2.7)
	*n (%)*
Female gender	163 (83.2)
Ethnicity/race	
African-American	87 (44.4)
Hispanic/Latino	22 (11.2)
Multi-ethnic/other	26 (13.3)
Non-Hispanic white	61 (31.1)
Education	
High school or less	23 (11.7)
Some college	67 (34.2)
College degree	62 (31.6)
Graduate degree	44 (22.5)
Income to poverty ratio	
0.00 – 1.99	74 (37.8)
2.00 – 3.99	56 (28.6)
4.00 – 5.99	31 (15.8)
≥ 6.00	35 (17.9)
Employment status	
Unemployed	44 (22.5)
Work disability	10 (5.1)
Part-time (<35 h/week)	38 (19.4)
Full-time (≥35 h/week)	85 (43.4)
Retired	19 (9.7)
Current smoker	22 (112)
	*Median (interquartile range)*
Body Mass Index (kg/m^2^)	29.9 (23.8, 36.3)
Daily energy intake from three 24-h recalls (kcal)	1912 (1523, 2367)

^a^Measured with “Short Form C” of the Marlowe-Crown Social Desirability Scale. Higher scores indicate greater social desirability

Over the 14-d assessment period, participants collected 2229 total food receipts (median = 10 receipts per participant) containing 16,356 line item food purchases (median = 75 purchased items per participant). Participants reported spending an average of 42.6 mins (SD = 48.9 mins) collecting and annotating food receipts over the 14-day assessment period, with 90% of participants requiring less than 100 min.

For descriptive purposes, food purchase data are displayed by source and overall in Table 2. Across subjects, 66.2% of foods and beverages were purchased from stores, with smaller percentages from fast food/carryout (19.4%), restaurants, taverns, and cafeterias (11.3%), and “other” food sources (3.1%). Neither HEI-2010_consumed_ (*ρ* = -0.08, *p* = 0.24) nor HEI-2010_purchased_ (*ρ* = -0.11, *p* = 0.11) was associated with social desirability score.

**Table 2:** Median (interquartile range) nutrient densities and diet quality for food purchases from different food sources and 24-h diet recalls (*N* = 196 participants; obs = 16,356 purchases)

	Overall *N* = 196; obs = 16,356	Store *n* = 196; obs = 10,826	Fast food/carryout *n* = 196; obs = 3169	Restaurant^a^ *n* = 196; obs = 1847	Other^b^ *n* = 196; obs = 507	24-h diet recalls *n* = 196
Receipts per participant^c^	10 (6, 15)	6 (4, 9)	3 (1, 6)	2 (1, 3)	1 (1, 2)	--
Purchases per participant^c^	75 (45, 110)	49 (27, 80)	14 (6, 28)	14 (7, 25)	3 (2, 12)	--
Purchases per receipt	7.0 (5.0, 10.3)	8.0 (5.0, 12.5)	4.6 (2.8, 6.5)	7.0 (4.0, 10.0)	2.0 (2.0, 6.0)	--
Total food mass (kg over 2 weeks)	34.5 (19.3, 56.5)	29.4 (15.0, 51.2)	2.3 (1.0, 4.4)	1.9 (1.0, 3.3)	1.1 (0.3, 6.7)	--
Total energy (kcal over 2 weeks)	45453 (21489, 76963)	36531 (16864, 65478)	3383 (1691, 6453)	2970 (1619, 4661)	3383 (1691, 6453)	--
HEI-2010	60.9 (49.1, 71.7)	59.4 (46.7, 72.6)	44.4 (38.0, 52.7)	47.6 (39.5, 57.9)	55.0 (33.4, 61.1)	60.1 (50.8, 73.9)
Energy density-food (kcal/g)	2.0 (1.7, 2.4)	2.0 (1.6, 2.6)	2.3 (1.8, 2.6)	2.0 (1.6, 2.4)	1.4 (0.3, 3.6)	1.7 (1.4, 2.1)
Energy density-beverages (kcal/g)	0.4 (0.3, 0.5)	0.4 (0.3, 0.6)	0.4 (0.1, 0.5)	0.4 (0.1, 0.6)	0.4 (0.3, 0.6)	0.1 (0.1, 0.2)
Fat (g/1000 kcal)	41.0 (35.3, 48.1)	41.6 (32.4, 48.8)	45.0 (39.1, 50.8)	43.0 (38.9, 49.6)	23.5 (10.5, 39.2)	40.7 (35.7, 44.8)
Saturated fat (g/1000 kcal)	13.1 (11.0, 15.3)	12.8 (10.5, 15.5)	14.4 (11.0, 16.8)	14.7 (10.9, 16.7)	3.9 (1.6, 14.3)	12.7 (10.8, 14.7)
Carbohydrate (g/1000 kcal)	118.9 (103.6, 139.1)	122.4 (103.0, 147.2)	109.0 (91.4, 123.1)	97.6 (78.6, 108.9)	170.1 (131.1, 218.5)	118.0 (104.5, 133.9)
Protein (g/1000 kcal)	34.7 (29.2, 41.4)	33.0 (26.0, 40.6)	40.7 (32.5, 48.8)	42.9 (35.3, 49.2)	39.3 (16.6, 62.3)	38.7 (33.9, 46.8)
Sugars (g/1000 kcal)	50.9 (40.0, 65.2)	54.6 (41.7, 73.0)	29.7 (14.1, 48.1)	22.1 (12.1, 32.1)	82.9 (30.7, 116.2)	48.0 (38.4, 61.9)
Sodium (mg/1000 kcal)	1343 (1064, 1811)	1252 (923, 1755)	1937 (1496, 2337)	1902 (1506, 2422)	598 (217, 1037)	1545 (1365, 1760)
Fiber (g/1000 kcal)	8.6 (6.0, 12.0)	8.7 (5.7, 12.3)	6.4 (5.0, 8.6)	6.8 (4.3, 9.3)	15.1 (7.0, 66.7)	9.9 (6.8, 12.9)
Whole fruit (cups/1000 kcal)	0.2 (0.1, 0.5)	0.3 (0.1, 0.6)	0.0 (0.0, 0.1)	0.0 (0.0, 0.1)	0.0 (0.0, 0.2)	0.2 (0.1, 0.5)
Vegetables (cups/1000 kcal)	0.6 (0.4, 1.0)	0.6 (0.2, 1.0)	0.7 (0.4, 1.2)	08 (0.4, 1.3)	0.7 (0.0, 18.0)	0.8 (0.5, 1.2)

^a^Category includes purchases from restaurants, bars/taverns, and cafeterias

^b^Category includes vending machine purchases, home-produced foods (e.g., vegetable gardens), mail ordered foods, and food sources marked as “other”

^c^Except for the “Overall” column, these values were derived among the subset of participants reporting at least one receipt/purchase from a given food source

**Fig. 2:**
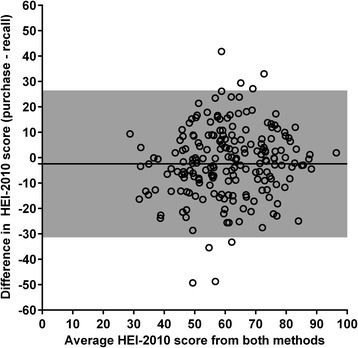
Bland-Altman plot depicting agreement between Healthy Eating Index-2010 scores derived from 24-h diet recalls and food purchase receipts (*N* = 196). Plot shows the mean difference between methods (bias) and 95% confidence intervals of the differences (shaded regions) across the distribution of scores (*N* = 196)

Concordance between HEI-2010_consumed_ and HEI-2010_purchased_ was moderate (ρc = 0.57, *p* < .0001). Bland-Altman analysis indicated that HEI-2010_purchased_ underestimated HEI-2010_consumed_ by 2.0 points on a 0–100 scale (Fig. 2). Concordance and bias were similar when HEI-2010_purchased_ was scored excluding beverages (ρc = 0.56; bias = -2.5) or cooking and baking ingredients (ρc = 0.60; bias = -0.9), and when the contributions of any individual food or beverage purchase was constrained to 5000 kcal (ρc = 0.58; bias = -1.7).

**Fig. 3:**
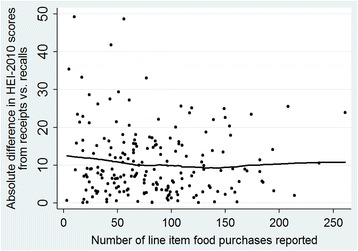
Lowess plot depicting a relatively consistent level of agreement between HEI-2010 scores calculated from purchase and diet recall data (*N* = 196), regardless of the number of line item food purchases reported

Discrepancy between HEI-2010_consumed_ and HEI-2010_purchased_ for each subject, quantified as a difference score, was unrelated to household size (ρ = 0.08, *p* = 0.24), income to poverty ratio (ρ = 0.04, *p* = 0.57), social desirability score (ρ = -0.06, *p* = 0.41), and BMI of the primary food shopper (ρ = 0.06, *p* = 0.39). Similarly, no associations were observed between these variables and the absolute values of difference scores (all ρ’s < |0.03|). The total number of food items reported by each subject was not associated with the absolute difference in HEI-2010 scores (*ρ* = -0.04, *p* = 0.54), and as shown in the Lowess plot in Fig. 3, agreement between HEI-2010 scores based on diet recall and purchase data was relatively consistent regardless of the number of line item food purchases reported.

Concordance correlations involving nutrient densities for purchased and consumed foods were lower for total fat (ρc = 0.10, *p* = 0.17), saturated fat (ρc = 0.17, *p* = 0.02), and sodium (ρc = 0.15, *p* = 0.04), and higher for carbohydrate (ρc = 0.24, *p* < 0.001), protein (ρc = 0.31, *p* < 0.0001), sugar (ρc = 0.28, *p* = 0.0001), fiber (ρc = 0.61, *p* < 0.0001), whole fruit (ρc = 0.48, *p* < 0.0001), and vegetables (ρc = 0.39, *p* < 0.0001).

## Discussion

The principal finding from this study is that diet quality scores derived from objectively documented household food purchases demonstrate moderate agreement with those derived from 24-h diet recall data. Estimates were unbiased across the distribution of scores, and did not vary by social desirability, BMI of the primary shopper, household income, or household size. Though there was great variability in the number of purchases reported by subjects, this did not impact agreement with diet quality. Observed agreement was also unaffected by the application of different scoring procedures designed to minimize the impact of beverages, cooking and baking ingredients, and bulk purchases on estimates. It can be concluded that food purchases, based on 2 weeks of receipt data, provide a reasonable estimate of the shoppers’ overall diet quality.

In contrast to the findings with HEI-2010 scores, agreement between food purchases and diet recall data was modest for several individual nutrient densities. This may stem from the fact that individual nutrient densities can vary substantially across days, whereas the HEI-2010 may be a more reliable value because it is an aggregate of 12 component scores. Additionally, the fact that HEI-2010 scores are scaled to a range of 0-5, 0–10, or 0–20 points per nutrient may increase agreement between purchases and dietary intake by truncating the range of scores (i.e., limiting extreme nutrient values to the top or bottom of the scoring range). Future research should explore the extent to which agreement between nutrient densities for food purchases and dietary intake increases when dietary intake is estimated from a greater number of 24-h diet recalls and food purchases are assessed over longer time periods.

It is important to note that food purchases and dietary intake would not be expected to demonstrate near-perfect agreement, even in theory. The upper bound of potential agreement is limited by food waste, consumption of foods that were not purchased by the individual, and the passage of time between the purchase and consumption of individual foods. Even if individuals personally purchased all of the foods that he or she consumed, each diet recall would represent only a “sample” drawn from the larger set of purchased foods available for consumption, and no diet recall would exactly represent the nutrient content of the entire set of purchases. Agreement is also affected by measurement error intrinsic to 24-h diet recall data, which includes recall and social desirability bias [42]. Though diet recalls do not yield valid estimates for energy intake, they can be used to estimate intake of other dietary components when energy intake is adjusted for [42]. The HEI-2010 derives diet quality scores based on nutrient densities (i.e., nutrients per 1000 kcal consumed), and thereby evaluates dietary composition independent of quantity. This property would be expected to reduce bias, and facilitate agreement between HEI-2010 scores for purchases and dietary intake. However, to fully characterize the impact of bias, it would be valuable to compare nutrient densities from food purchases and diet recalls with objective nutritional biomarkers.

Over two-thirds of reported purchased foods were obtained from food stores. HEI-2010 diet quality scores were 12–15 points lower (on a 100-point scale) for food purchases at fast-food and full-service restaurants relative to purchases from food stores. Though we did not have adequate sample sizes within each food source to formally test these differences, the observed pattern corresponds with prior studies reporting differences in nutrient densities for packaged foods obtained from different store types [12], and for fast food menus relative to the larger U.S. food supply [11]. In fact, the difference in diet quality for purchases from food stores vs. restaurants is larger than the difference between the 50th and 75th percentiles of diet quality in the U.S. population [43]. Though no interpretive guidelines for HEI-2010 scores have been developed, diet quality generally exhibits a linear association with mortality and chronic disease risk [44], so reliance on fast-food and full-service restaurants would be expected to have implications for health [45].

The procedures used to collect, analyze, and score the nutrient content of food purchases could be used in future studies to assess the contribution of purchasing patterns to obesity and chronic disease risk, and examine changes in purchasing in response to dietary interventions. Though the food purchase documentation protocol has a relatively low participant burden, converting receipt data into nutrient data requires additional processing by research staff. Semi-automated methods such as Nielsen’s National Consumer Panel (HomeScan) and the UK’s Kantar World Panel would be more feasible for population-level monitoring. In contrast, receipt analysis protocols such as that used in this study would be valuable to researchers interested in assessing purchasing of non-packaged food items such as fresh produce and away-from-home meals, which are either captured unreliably or not at all with scanner-based documentation protocols [27, 28]. Additionally, the current protocol would enable research examining relations between food purchasing patterns and variables that are not available in large consumer panels, including various social and behavioral characteristics, medical outcomes, or exposure to interventions. As food purchases can be objectively documented, estimates of nutrient intake derived from them may be less susceptible to bias than those derived through 24-h diet recalls and food frequency questionnaires, though neither were correlated with social desirability in this study.

A key strength of this study was the application of a rigorous food purchase assessment protocol that included photographic documentation of purchases in participants’ homes. The study sample was socioeconomically and ethnically diverse, and focused on household members who made the majority of food purchases for their household. The observed HEI-2010_consumed_ score (M = 60.1) is similar to that in the broader U.S. adult population (M = 58.3) [46], which indicates a degree of external validity. However, as dietary intake was only assessed for the primary food shopper, the extent to which household food purchases represent dietary intakes for other household members requires further study. An additional limitation was that relatively few purchases were reported from certain sources (e.g., cafeterias), which reduced the precision in nutrient density estimates within these sources and precluded comparisons between food sources. It was not possible to derive nutrient data for some ready-to-eat foods with inestimable portion sizes (e.g., buffet meals, complex restaurant meals). Though this occurred somewhat infrequently, this limitation could meaningfully impact estimates of overall diet quality for individuals who heavily rely on buffets and similar food sources.

## Conclusions

Nutritional analysis of objectively documented food purchases yields a reasonable estimate of overall diet quality for primary household food shoppers, but accurately represent intake for only some individual nutrient densities. Though cautious interpretation is warranted, the collection and analysis of receipts may enhance research on the role of household food purchasing patterns in diet quality and chronic disease risk.

## Abbreviations

BMI: Body mass index
HEI-2010: Healthy eating index-2010
NDSR: Nutrition data system for research
